# Complete genome analysis of *Edwardsiella tarda* strain MYK24826 isolated from a Japanese patient with prosthetic valve endocarditis

**DOI:** 10.1128/mra.01443-25

**Published:** 2026-04-29

**Authors:** Masayuki Imajoh, Rina Murakami, Jotaro Abe, Noriyuki Abe, Yoshiaki Cho, Masashi Narita, Noriko Ohyama

**Affiliations:** 1 Department of Marine Resource Science, Faculty of Agriculture and Marine Science, Kochi University 12888, Kochi, Japan; 2 Division of Infectious Diseases, Department of Internal Medicine, Okinawa Prefectural Nanbu Medical Center and Children’s Medical Center, Haebaru, Japan; 3 Department of Cardiovascular Surgery, Okinawa Prefectural Nanbu Medical Center and Children’s Medical Center, Haebaru, Japan; 4 Department of Pediatric Infectious Diseases, Okinawa Prefectural Nanbu Medical Center and Children’s Medical Center, Haebaru, Japan; 5 Division of Cardiovascular Surgery, Department of Surgery, Kobe University Graduate School of Medicine 593152, Kobe, Japan; Loyola University Chicago, Chicago, Illinois, USA

**Keywords:** *Edwardsiella tarda*, Japanese patient, prosthetic valve endocarditis

## Abstract

The complete genome sequence of the *Edwardsiella tarda* strain MYK24826, isolated from a Japanese patient with prosthetic valve endocarditis, was determined. The genome consisted of a 3,647,674 bp circular chromosome and a 2,866 bp plasmid. The genotype of MYK24826 was identified as sequence type ST28 through multilocus sequence typing.

## ANNOUNCEMENT

*Edwardsiella tarda*, a member of the family *Enterobacteriaceae*, is commonly found in aquatic environments, which explains its relatively infrequent involvement as a human pathogen (1). Although gastroenteritis represents the most typical presentation of *E. tarda* infection, extraintestinal diseases, including bacteremia, have also been documented (1–3). In Japan, the widespread presence of brackish-water habitats and river systems, combined with substantial consumption of raw seafood that may harbor *E. tarda*, is considered a contributing factor to the risk of infection (1).

Strain MYK24826 was recovered from the blood of a Japanese patient diagnosed with prosthetic valve endocarditis. Isolation was performed after overnight incubation on sheep blood agar/Drigalski modified agar (Shimadzu Diagnostics Corporation, Tokyo, Japan) and on Chocolate II agar (Becton Dickinson, Tokyo, Japan) at 35°C under 5% CO₂. The isolate was identified as *E. tarda* by matrix-assisted laser desorption/ionization time-of-flight mass spectrometry (MALDI-TOF MS) (4) and subsequently stored at −30°C until analysis. The study was approved by the Okinawa Prefectural Nanbu Medical Center Ethics Committee (Approval No. R7-173), with written informed consent obtained during surgical consent.

Frozen stock of MYK24826 was cultured on BHI agar (BD Difco, USA) overnight at 37°C. A single colony was then inoculated into 10 mL of BHI broth and incubated with shaking overnight at 37°C. After centrifugation at 16,000 × *g* for 2 min, genomic DNA was extracted from the bacterial pellets using the Wizard HMW DNA Extraction Kit (Promega, USA) and used for both Illumina and Nanopore sequencing. For Illumina sequencing, genomic DNA was enzymatically fragmented to an average size of 330 bp and processed with the QIAseq FX DNA Library Kit (Qiagen, USA) according to the manufacturer’s instructions. After adapter ligation, 20 pM pooled libraries were sequenced on an Illumina MiSeq (MiSeq Reagent Kit v3; 150 cycles, USA), producing 75 bp paired-end reads. MiSeq raw reads with Phred quality scores above 30 were retained for assembly using Trim Galore v.0.6.5 (5). For nanopore sequencing, genomic DNA was prepared using the 1D Ligation Sequencing Kit (SQK-LSK109, Oxford Nanopore Technologies, USA) with Native Barcoding Expansion (EXP-NBD104), omitting shearing and size-selection steps to preserve fragment integrity. Following adapter ligation, pooled libraries were sequenced on a MinION with an R9.4.1 flow cell using MinKNOW v22.05.5. Base calling was conducted with Guppy v.4.2.3 in accurate mode. MinION reads with minimum quality scores below eight were removed using NanoFilt v.2.8.0 (6). Unicycler v.0.4.8 (7) was used for the hybrid assembly of Miseq and MinION reads. Genome error correction, circularization, and rotation were performed within the Unicycler workflow. Gene annotation, taxonomy confirmation, genomic average nucleotide identity (gANI) analysis, and genotyping were performed using the DDBJ Fast Annotation and Submission Tool v. 1.2.0 (8) and the *E. tarda* MLST databases (9), with default parameters applied unless stated otherwise.

The genomic features of MYK24826 are summarized in Table 1. MLST identified MYK24826 as sequence type ST28, which is distinct from ST25 of the previously reported *E. tarda* strain GBS0709 with a complete genome sequence (10).

**TABLE 1:** Genome characteristics of *E. tarda* strain MYK24826

Feature	Data
Number of contigs	2
Structure	Circular chromosome and plasmid
Number of Miseq reads	1,531,054
Number of MinION reads (N50 value (bp))	85,418 (7,685)
Total length (bp)	3,650,540
GC content (%)	57.3
Coverage (×)	136
N50 value (bp)	3,647,674
Number of coding sequences	3,316
Number of rRNAs	28
Number of tRNAs	102
Whole-genome sequence accession no.	AP040123 for the chromosome and AP040124 for the plasmid
DRA accession no.	DRR725188 for Illumina MiSeq data and DRR725189 for MinION data
gANI values of MYK24826 for *E. tarda* strains	
ATCC15947 (GCA_000264805.1)	99.43
ATCC15947 (GCA_003113495.2)	99.28
FDAARGOS_1473 (GCA_019933175.1)	99.28
NBRC105688 (GCA_000341505.1)	99.28

## Data Availability

The complete genome sequence of *E. tarda* MYK24826 is available at National Center for Biotechnology Information under accession numbers AP040123 (chromosome) and AP040124 (plasmid). Raw sequence data from Illumina MiSeq and MinION are available at DDBJ Sequence Read Archive under DRA accession numbers DRR725188 and DRR725189.
